# Investigating the impact of ambulance service models on paramedic delivery of end-of-life care in England: an exploratory study using case study design

**DOI:** 10.1186/s12904-026-02283-x

**Published:** 2026-08-12

**Authors:** Natasha Campling, Sydney Anstee, Sue Latter, Alison Richardson, Joanne Turnbull, Jennifer Scott-Green, Sarah Voss

**Affiliations:** 1 School of Health Sciences, University of Southampton, Southampton, UK; 2 South East Coast Ambulance Service NHS Foundation Trust, Crawley, UK; 3 School of Health and Social Wellbeing, University of the West of England, Bristol, UK

**Keywords:** Palliative care, Terminal care, Paramedics, Emergency medical services, Ambulances, Case study, Qualitative research

## Abstract

**Background:**

Rising demand for end-of-life care in developed countries, driven by ageing populations and multimorbidity, has led to increased calls to emergency ambulance services. Despite this little is known about paramedic practice in this context and service provision varies. As part of a wider study evaluating paramedic delivery of end-of-life care, in-depth case studies were undertaken to investigate how different models of ambulance service shape paramedic practice and influence experiences.

**Methods:**

Embedded case study design. The unit of analysis for each case comprised a distinct service model. Three ambulance services in England, UK were selected for their end-of-life service specifications. Semi-structured interviews and focus groups were undertaken with patients, family carers and healthcare professionals and data extracted from ambulance patient records. Qualitative data were analysed using Framework Analysis and patient ambulance record data using descriptive statistics and directed content analysis. Data were collected in parallel and analysed together utilising a mixed method, convergent parallel design. Integration occurred initially within cases, then across the cases.

**Results:**

Three cases were studied. They represented a usual model of end-of-life care delivery (Case 1) and more innovative models (Cases 2 & 3). Anticipatory medicines were carried by advanced paramedics in Case 2, and Case 3 had an in-service specialist palliative care team and access to palliative care ambulances. One hundred and one (*n* = 101) individuals participated, including *n* = 75 healthcare professionals and *n* = 26 patients and carers. In all cases, paramedics worked hard to provide supportive care, often attending due to primary and community service shortages. Learning on the job and limited access to essential resources (patient healthcare records, anticipatory medicines and/or palliative care advice) dominated practice across the cases. Access to advanced paramedics who carry anticipatory medicines in Case 2 and palliative care ambulances in Case 3 facilitated care provision and improved patient outcomes (death in preferred place).

**Conclusions:**

This study provides an in-depth exploration of paramedic practice at end-of-life via case studies of service models. To best support paramedics in practice, services should facilitate end-of-life care training, access to and ability to administer medicines, and promote interdisciplinary collaboration, underpinned by access to shared digital patient healthcare records.

**Supplementary Information:**

The online version contains supplementary material available at https://doi.org/10.1186/s12904-026-02283-x.

## Background

Most countries have some form of emergency medical service but globally, services are highly heterogeneous [1]. Developed nations typically have established, government-regulated ambulance systems while in developing regions infrastructure and communication limitations may restrict services and effectiveness of emergency healthcare [2]. The UK has the world’s longest established emergency helpline (999), introduced in 1937 [3]. Following 999 call handling triage, ambulance crews attend the patient. Crews are usually led by paramedics, following the Anglo-American model of provision where paramedics act as the main clinical providers of care (as opposed to medical doctors who do so in the Franco-German model) [4]. England’s ambulance service is provided by 11 National Health Service (NHS) trusts [5]. Such provision by multiple providers can enable variation in ambulance service care to exist, which may affect the quality of all emergency care and impact patient and family experiences. Lord Coles’ review (2018) of operational productivity and performance in English NHS Ambulance Trusts found significant and increasing disparity between trusts, particularly in the models of care provided to patients [6]. It is known that service variation such as the availability of alternative care pathways, results in different non-transport (non-conveyance to Emergency Department) rates between ambulance services [7–10]. But what is not known, is how these service variations - that form the context within which care is delivered - impact care delivered to people who are at the end-of-life (the last year of life). Variations in this specific context include availability of specialist palliative and end-of-life care expertise (dedicated teams) within trusts and availability of anticipatory medicines (often termed just-in-case medicines) carried by specialist-level paramedics with the authority to administer. These medicines are administered sub-cutaneously for rapid symptom management, typically of pain, nausea/vomiting, anxiety/agitation, respiratory secretions and breathlessness [11]. This international evidence gap is intensified by limited research about patient and family carer experiences of frontline paramedic involvement at end-of-life [12–14].

In the UK emergency ambulance attendances and conveyance rates for people in the last year of life are substantial [15, 16]. In a 2023 survey of bereaved carers across England & Wales, 645 of 1,179 (55%) of people who died had used an ambulance at least once in the last three months of life [17]. In Scotland [18] and in Wales [19] 77% and 80% of people living at home in their last year of life phoned the ambulance service. Tackling avoidable conveyances to hospital and supporting delivery of care where possible at home, would reduce unnecessary pressures on Emergency Departments and release capacity equivalent to £300 million [6, 16]. Rates of ambulance service use and associated health service costs are anticipated to further increase [16], with rising average age of death, increasing frailty, comorbidities and palliative and end-of-life care needs [20, 21].

Our previous research revealed that frontline paramedics in England attending patients at home in the last year of life reported challenges to delivering care [22]. These include fear of taking the wrong course of action, encountering family conflict, a lack of access to patient records and medicines and a lack of availability of healthcare professionals for advice and referral especially during out-of-hours periods [22]. Challenges to the delivery of care can mean people have less effective symptom control, increased distress and/or may be unnecessarily transferred to hospital when they could be more appropriately cared for at home, in accordance with their stated wishes and preferences [23–25].

Therefore, as part of a wider study evaluating paramedic delivery of end-of-life care (funded by Marie Curie MC-21-802) the objective for this study was to:

- Understand how different models of ambulance service provision shape paramedic practice and influence experiences for individuals at end-of-life.

The study is registered at Open Science Framework: https://doi.org/10.17605/OSF.IO/4MP5K.

## Methods

### Design

Embedded design case studies were undertaken utilising Yin’s approach [26]. Yin’s approach was selected, an approach widely used within health services research, facilitating explanatory research, utilising multiple sources of evidence (both qualitative and quantitative) across multiple cases [27, 28].

The unit of analysis for each case (*n* = 3) was a distinct service model, each with different end-of-life related service specifications (Table 1). The cases were bounded by the service boundaries (organisational and geographical) of each ambulance service, as limiting cases to teams within services for example would have led to blurred boundaries and unbounded cases. Data collection methods primarily utilised qualitative methods, supported by a secondary method of patient record data extraction. The patient record and interview data were nested within the cases (embedded design). Data were collected in parallel and analysed together utilising a mixed method, convergent parallel design. Integration began once data had been collected across the three cases. This process was underpinned by case study design principles of triangulation of data sources via sample group initially within each case, followed by comparative analysis across the cases [26]. Embedded design logic was followed, with both the qualitative and quantitative data informing each other [26]. The Standards of Reporting Qualitative Research (SRQR guideline) has been used to report the study [29].

### Sampling

Service models were purposefully sampled following our earlier large-scale online survey across all English ambulance services [22]. These data enabled purposeful sampling of service models (ambulance services) that encompassed both established (usual) models of end-of-life care delivery and more innovative models (Table 1).

**Table 1:** End-of-life related service specifications in the case studies

Case 1 - Usual service model	Case 2 – Anticipatory medicines carried by advanced paramedics	Case 3 – In-service specialist palliative care team + palliative care ambulances
• No palliative or end-of-life care service specifications/features	• End-of-life care service lead • Anticipatory (just-in-case) medicines carried by advanced paramedic practitioners, for use via Patient Group Direction (PGD) at end-of-life: cyclizine, midazolam and hyoscine butylbromide • End-of-life care in-service training • Advance care planning documents where shared with the ambulance service are saved within the service’s patient record system • Specialist palliative care placements for registered paramedics	• In-service specialist palliative care team (*n* = 3 specialist nurse, engagement officer + administrator), including end-of-life care service lead • End-of-life care in-service training • Advance care planning documents where shared with the ambulance service are saved within the service’s patient record system • Three (*n* = 3) “palliative care ambulances” - dedicated end-of-life care transfer vehicles (commissioned by the ambulance service, delivered by charitable ambulance provider), for transfer to preferred place of death e.g. hospital to home, hospice to home (not 999 response)

Case 1 was selected to represent an established, usual model of service delivery with no end-of-life related service specifications. Cases 2 and 3 were selected to represent ambulance services that had implemented innovative end-of-life related service specifications, distinct from one another.

Sample sizes within the case studies were determined by considerations of analytical generalisability and informational power within each case [30]. For each case, the anticipated sample size was *n* = 20 healthcare professionals and *n* = 10 patients and/or carers. Therefore, the overall target sample size was *n* = 90: *n* = 60 healthcare professionals, and *n* = 30 patients and/or carers.

### Data collection

Data collection involved the following recruitment routes and inclusion criteria (Table 2).

**Table 2:** Recruitment and inclusion criteria

	Recruitment	Inclusion Criteria
PATIENTS & CARERS	Sampled via n = 2–3 hospices selected due to being large and research active within the geographical footprint of the case and willing to participate. NIHR regional research delivery network clinical staff, clinical and research staff in the hospices screened hospice caseloads for eligible individuals. Repeat searches for new patients were conducted monthly. NIHR regional research delivery network clinical staff, clinical and research staff in the hospices screened hospice caseloads for eligible individuals. Repeat searches for new patients were conducted monthly.I n the last four months of recruitment, with an approved ethical amendment, ‘indirect advertising’ also occurred via participating organisations and groups including local Healthwatch and bereavement groups.	*Patients/carers*: Adult patients (18 and above) in the last year of life, receiving specialist palliative care, in receipt of service delivery from providers connected to respective case study and noted to have received emergency attendance by paramedics in the last few weeks/months (no more than 6 months). With capacity to consent to take part in the research.Carers of patients (meeting above eligibility) could take part either with the patient or on their own.Where recruitment was lower than planned, bereaved carers of individuals attended by paramedics in the last few weeks/months of an individual’s life were also included.
HEALTHCARE PROFESSIONALS	*General practitioners*: Expressions of interest to take part in an interview were distributed to research active practices via the NIHR regional research delivery network (mapped to the geographical footprint of the case). *Community specialist palliative care clinicians*: Invited to attend a focus group by key clinical staff in the participating hospices with the case study. *Community nurses*: Research and delivery staff within the largest community Trust connected to the case, identified local collaborators within the Trust cascading the invitation to participate in a focus group to community nursing teams. *Paramedics and ambulance technicians*: Invited to participate in a focus group and/or 1:1 paramedic vignette interview through advertising, via usual internal communications routes, distributed by the ambulance Trust’s research team.	Registered paramedics and ambulance technicians (non-registrants) employed in the ambulance trusts whose service model was being studied. Other healthcare professionals: GPs, community nurses and community based palliative care teams, who were the main providers of community-based end-of-life care in the respective case.

Data were collected between June 2024 and June 2025 and included varying data sources to capture multiple stakeholder perspectives.

### Patients/carers

Patients and/or carers (both current and bereaved) took part in semi-structured interviews. Interviews were one-off and conducted in person, online or via telephone as preferred, to explore experiences of paramedic delivered care. An interview guide was designed to focus on experiences in relation to no more than *n* = 3 ambulance attendances over recent months (to avoid recall issues). Patients were asked whether they consented to data extraction from their ambulance records. A data extraction form was developed in consultation with the research teams in the respective ambulance services, based on ambulance electronic patient record fields. Extracted data focused on care processes and outcomes: clinical impression on scene including care required for family members, administration of medicines for symptom control, and how supplied, conveyance to hospital or not, onward referrals, time on scene and if repeat 999 calls/recontacts were made.

Potential participants were given information packs (invite letter, participant information sheet and consent form) by their usual specialist palliative care provider for patients/carers, or their employing organisation/clinical team lead for health professionals. Written informed consent was obtained for all participants by the study’s senior research fellow (SA).

### Healthcare professionals

Paramedics (registered ambulance clinicians) and ambulance technicians (non-registered ambulance clinicians) took part in online focus groups and asked to outline their practices and experiences of providing end-of-life care. Other healthcare service professionals participated in online, face-to-face semi-structured interviews (general practitioners) and focus groups (community nurses and specialist palliative care clinicians). Semi-structured interviews and focus groups (guided by two facilitators with *n* = 4–8 participants sought per group) were held to explore their experiences of paramedic delivered end-of-life care, including barriers and facilitating factors. All topic guides were developed by the research team in consultation with the study’s advisory group (see supplementary file 1 – topic guides).

Paramedics also took part in 1:1 online vignette interviews where ‘real-life’ end-of-life vignettes were used to explore paramedic decision-making at end-of-life, including how risk was assessed and managed [31, 32] (see supplementary file 2 for details of the vignettes). Findings from the paramedic vignette interview data are included here, but will be the focus of a further publication, as the research objective for these data focused on explaining paramedic decision-making.

### Analysis

Semi-structured interviews and focus groups were audio-recorded, fully transcribed and analysed using Framework Analysis [33]. The five-stage process of data familiarisation, identification a thematic framework, indexing data against the framework, charting to summarise the indexed data, and mapping and interpretation of patterns found within the charts was followed [33]. Frameworks were developed iteratively (NC & SA) for the general practitioner, community nursing, paramedic and ambulance technician, specialist palliative care clinician and patient/carer datasets. SA initially coded the datasets, followed by collective review and discussion within data analysis workshops (NC, SA, AR, JT, SL). Paramedic vignette interview data were included in the respective case datasets and in case analysis where data influencing responses were specific to the nature of the case (the service model).

Patient ambulance record data were analysed using directed content analysis [34]. Data within the data extraction forms were analysed using this method and directed towards: events leading to the attendance, clinical impression on scene, nature of care provided including administration of medicines for symptom control, advice/further consultation sought, conveyance, onward referrals and outcome. NC led analysis of these data, supported by JSG and a patient record data analysis workshop was held to discuss these data, integrating them with the corresponding patient/carer interview data (NC, JSG, SA).

Data were triangulated via data source within each case, followed by cross-case comparison exploring the similarities and differences (pattern matching) across the cases to support explanation building and analytical generalisability [30]. Systematic comparisons explored how the features of the services facilitate or preclude paramedic provision of end-of-life care. Data integration occurred at the level of the case. Firstly, data were charted using Framework Analysis by data source: patient/carer interviews, specialist palliative care clinician focus group, community nurse focus group, paramedic and ambulance technician focus group, and general practitioner interviews. Secondly, case specific data from the paramedic vignette interviews was extracted. Thirdly, the patient record data extraction was summarised as a dataset using directed content analysis (focused on patient history, episode of care and actions undertaken). Fourthly, data from all data sources were compared within each case.

### Research team and reflexivity

Data were collected within the cases by SA (an experienced researcher), supported and supervised by NC as study lead. The study team (NC, SA, SL, AR, JT, JSG, SV) met monthly during the study, with additional data analysis workshops undertaken. Study team members NC, SL and JT (each with expertise in case study methodology) led data analysis of a case, prior to cross-case analysis. Data analysis benefited from extensive clinical/practice and academic expertise in the following areas: paramedicine, palliative and end-of-life, emergency care, health services research and medicines management; and methodological expertise in case studies and mixed methods. Research rigour and objectivity were ensured via team meetings and workshops, and the use of field notes and carefully designed data collection tools.

### Ethical approval

Ethical approval was obtained via the University of Southampton Faculty of Environmental and Life Sciences (ERGO reference: 90972), Health Research Authority (IRAS reference: 340051) and NHS Research Ethics Committee – London Surrey (REC reference: 24/PR/0405) and subsequent organisational governance approvals gained.

### Participants and data sources

Table 3 displays participant recruitment and Table 4 associated data sources for each case. Overall, 101 participants took part (*n* = 75 healthcare professionals, *n* = 11 patients and *n* = 15 carers). Demographic details for all participants can be found in supplementary file 3 (participant characteristics by case).

**Table 3:** Participants by case

CASE 1 – Usual service delivery	
Patients	6
Carers (*n* = 2 current carers, *n* = 2 bereaved carers)	4
Community specialist palliative care clinicians (*n* = 1 medical doctor, *n* = 1 nurse, *n* = 1 occupational therapist, *n* = 1 pharmacist)	4
Community nurses	6
General practitioners	2
Paramedics	10
Ambulance technicians (non-registrants)	2
Case 1 participant total	**34**
CASE 2 - Anticipatory medicines carried by advanced paramedics	
Patients	4
Carers (*n* = 3 current carers, *n* = 6 bereaved carers)	9
Community specialist palliative care clinicians (*n* = 1 medical doctor, *n* = 1 paramedic, *n* = 3 nurses)	5
Community nurses	5
General practitioners	2
Paramedics	14
Ambulance technicians (non-registrants)	2
Case 2 participant total	**41**
CASE 3 - In-service specialist palliative care team + palliative care ambulances	
Patients	1
Carers (*n* = 1 current carer, *n* = 1 bereaved carer)	2
Community specialist palliative care clinicians (*n* = 2 medical doctors, *n* = 2 nurses)	4
Community nurses	5
General practitioners	2
Paramedics	10
Ambulance technicians (non-registrants)	2
Case 3 participant total	**26**

**Table 4:** Data sources by case

CASE 1 – Usual service delivery	
Patient/carer interviews (*n* = 4 patient interviews, *n* = 2 patient + carer dyad interviews, *n* = 2 bereaved carer interviews)	8
Healthcare professional interviews/focus groups (*n* = 2 GP interviews, *n* = 1 community specialist palliative care clinician focus group, *n* = 1 community nurses focus group, *n* = 1 paramedic/ambulance technician focus group)	5
Paramedic vignette interviews	6
Patient record data extractions	6
CASE 2 - Anticipatory medicines carried by advanced paramedics	
Patient/carer interviews (*n* = 1 patient interview, *n* = 3 patient + carer dyad interviews, *n* = 6 bereaved carer interviews)	10
Healthcare professional interviews/focus groups (*n* = 2 GP interviews, *n* = 1 community specialist palliative care clinician focus group, *n* = 1 community nurses focus group, *n* = 1 paramedic/ambulance technician focus group)	5
Paramedic vignette interviews	6
Patient record data extractions	4
CASE 3 - In-service specialist palliative care team + palliative care ambulances	
Patient/carer interviews (*n* = 1 patient interview, *n* = 1 current carer interview, *n* = 1 bereaved carer interview)	3
Healthcare professional interviews/focus groups (*n* = 2 GP interviews, *n* = 1 community specialist palliative care clinician focus group, *n* = 1 community nurses focus group, *n* = 1 paramedic/ambulance technician focus group)	5
Paramedic vignette interviews	6
Patient record data extractions	1

## Results

Results are presented sequentially for each case study, followed by cross-case analysis.

### Case 1 – Usual service model

In the usual service model, patient and carer experience was one of multiple callouts (the highest of all the cases), exacerbated by lack of input from community-based services. Perhaps because of the frequency of callout episodes and lacking community service provision patients and carers were often extremely complementary of the care they received from ambulance crews. Healthcare professional perspectives varied of the paramedic role at end-of-life demonstrating a lack of common understanding, combined with limited paramedic training in palliative and end-of-life care leading to shortfalls in expertise, which precluded shared professional decision-making in this case.

#### Patient and carer experience

##### Context and nature of callouts

Patients and carers discussed at least two to three callout episodes (999 call and ambulance attendance) during interviews. Patient ambulance record data extraction demonstrated all patients (*n* = 6) had between two and 15 (mean 7.8, standard deviation 4.7) callouts in the six-month period prior to the most recent callout episode discussed at interview. Repeated callout episodes were required because of the fluctuating nature of underlying conditions and responses to treatment such as chemotherapy induced neutropenia. Patient experience also suggested that frequent callouts, at times on the same day or consecutive days, were because of failed hospital discharges:

“…The other one was about a fortnight before. I had them out twice in one day (2 callout episodes). Took me in in the morning and then I had the same trouble in the afternoon, they had to take me in… they came out in the morning because I had a bad turn, brought me back home on the same day, and they had to come out again later on in the day to take me back again…” (Patient-07-Case1).

Patient and carers described a lack of planned care elsewhere in the health service contributing to ambulance callouts particularly with shortcomings in general practice and community nursing services. However, significant unmet need from all community-based services, including specialist palliative care, meant callouts were perceived as occurring due to a lack of pre-emptive care:

“…All [hospital haematology, community nursing service, hospice service] said to us: “ring us if you need us”, but they didn’t come very easily, anybody, if I’m going to be honest… [Hospice] their visits had been few and far between and, we certainly felt the impact of what sort of now is nationally recognised that a lot of hospices are struggling financially and reducing their services, and we had district nurses. But, I think, even in the worst week we had, they only came in once a week…” (Bereaved carer-04-Case1).

Patients and carers were repeatedly advised to call 999 by healthcare professionals. Those receiving palliative chemotherapy and at risk of neutropenia had urgent sepsis cards and knew to contact their oncology or haematology team, at the first sign of infection. Three participants spoke about their teams telling them to call 999 and what to say on the call. Other examples of healthcare professionals advising patients or carers to call 999 were also provided:

“…I’d ‘phoned (hospice) because my first of call is (hospice). So, I spoke to somebody at (hospice) and obviously, in the middle of the night, they have got nobody there really that can come out so, they just told me to ‘phone 999 because he was in so much pain. So, we did because we didn’t know— we’d given him everything that we could…” (Patient + carer-08-Case1).

##### Care from ambulance crews perceived as supportive

Patients and carers commonly praised the care they received from ambulance crews. They referred to feeling supported and listened to, with none sensing the crews needed to be elsewhere even when queuing outside hospital. They reported being asked about their wishes and what they would like to happen, and that assessments were clearly explained. There were also descriptions of human touches that made a difference to patients and their families in distressing circumstances:

“…They were fantastic, very reassuring, very caring, asking about me all the time, checking on me, I did feel very looked after…I think, one of the ambulance staff may have passed their mobile on to [my partner] to say: “this is where we are [outside the hospital] come here to her… They explained the situation to me and what they would do, and they said that they would be with me until I got took through and they didn’t know how long that would be… Their care and attention and their due diligence of what they were doing was a similar experience to the last time… they were so supportive, so caring, they were very, very professional…” (Patient-03-Case1).

#### Healthcare professional perspectives

##### Varied perception of paramedic role

There were variable perceptions of the paramedic role at end-of-life, often with limited understanding of evolved scopes of practice particularly on the part of general practitioners.

“…There’s a massive misunderstanding within other areas of healthcare as to what we do, and what our scope of practice is… GPs potentially aren’t as aware as to what our scope is and that we don’t just put people on an ambulance and drive them in anymore (laughing), that we do actually treat them and care for them in the community as well…” (Paramedic and technician focus group-Case 1).

“…I think the role of paramedics and ambulance services is limited in the care that paramedics can provide…” (General practitioner1-Case1).

For other healthcare professionals there was clear recognition of their significant role and what this required in terms of paramedic skills to help patients to remain at home if appropriate.

“…From an historical point of view, the ambulance service have been go, see a patient, pick them up and take them to hospital. We’re now asking them to work differently to that and to actually [think]: “What can we do?” What can they do? They’ve got all these skills so how can they utilise their skills to keep those patients at home…?” (Community nurse focus group-Case 1).

##### Ambulances called due to shortfalls in primary and community-based services

Participants from all sample groups expressed the view that if community-based care was effective then the need for ambulance callouts would be limited. For example:

“…If things are handled properly, it should be very rare that an ambulance is called in if someone is end-of-life because there should be a plan in place. There should be district nurses and perhaps a palliative care team involved as well as a GP practice…” (General practitioner2-Case1).

However, limits to the availability of primary and community-based services were obvious in this case and participants recognised that paramedics and the ambulance service had become a “*catch-all*” service as a result. Participants highlighted shortfalls in general practice and community nursing services leading to callouts. Lack of home visits by general practitioners were cited, as well as general practitioners themselves advising patients and carers to call 999:

“…There’s a large number [of general practitioners] now [since Covid] that don’t go out and review their patients, and unfortunately, their first answer to a relative or a patient on the phone if they’re really, really unwell is: ‘call 999’ (laughing)…” (Community nurse focus group-Case 1).

Significant staffing shortages in community nursing and implications for ambulance service were much discussed:

“…[Community nursing has] lost a lot of staff over the years and especially of a weekend. Sometimes, they might only have like one/two nurses on covering a huge area, and there is, one particular district nurse team that out of hours they just haven’t got the staff to work nights. So, they have now switched their service so, if somebody rings up, they say to the patients: ‘OK, from this point in time, we’re not covering so, you need to call an ambulance’.…” (Specialist palliative care focus group-Case 1).

Pressures on community services and resulting deficiencies in service provision leading to callouts were not just an issue out-of-hours, although this period was acknowledged as particularly problematic.

##### Limited training and palliative and end-of-life expertise hampers quality of care

Participants throughout the case recognised that paramedics had limited education and training in end-of-life care. Paramedics described a lack of continuing professional development:

“…We get typically sort of two training days a year, one is an online workbook, and one is a mandatory face-to-face training, but there’s already so many competing demands on it. The workbook is like twenty different chapters which don’t really go into detail on any of those but they’re things that the Trust consider their priorities at the moment, and the mandatory day probably will have about four themes across the day. So, to do any kind of justice to palliative care, I think, it would be a twenty-minute session on the end of a mandatory day which probably wouldn’t give us the kind of detail that we need to make these big decisions…” (Paramedic and technician focus group-Case 1).

General practitioners acknowledged paramedics had only “*basic training*” and associated this with paramedics not carrying anticipatory medicines in this case study site, as well as a lack of confidence to administer patient’s own anticipatory medicines.

“…I’m not aware of any specialism in that area [end-of-life care] from the point of view of the paramedics, but they have some general basic training in terms of how to manage it and they wouldn’t carry those [anticipatory] drugs themselves…” (General practitioner2-Case1).

Specialist palliative care clinicians questioned whether paramedics had any specific palliative and end-of-life care training because they perceived lack of expertise:

“…Do paramedics have any training in end-of-life care and palliative patients, and what to look for? I don’t know…” (Specialist palliative care focus group-Case 1).

Participants perceived the ambulance service and paramedics to lack clinical expertise in this area of care. This was a widely held view across the case amongst community-based professionals. Numerous descriptions were provided, for example lack of depth of assessment, challenges identifying and managing symptoms other than pain, difficulty recognising imminent end-of-life and palliative care emergencies, and appropriately distinguishing between what might be reversible and appropriately treated:

“…They [ambulance crews] don’t see what we would class as a palliative care emergency. They don’t see that as an emergency…” (Specialist palliative care focus group-Case 1).

“…It doesn’t surprise me if a paramedic sees that reversible cause would go to hospital with a reversible cause, and even if it is a drop in blood pressure because of the dying phase or whether they have got an infection, they’ll straight away just think: “well, it’s a reversible cause, we’ll take them in anyway…” (Community nurse focus group-Case 1).

Overall lack of expertise in the paramedic workforce was perceived to be due to lack of experience and a dominance of newly qualified paramedics (within two years of qualification) within the workforce:

“…Half the workforce have only been in post for two years, you know, where is that experience? And those kind of skills in learning those things and seeing patient after patient after patient and recognising that it’s patients that are as poorly as they are. I guess, that is going to be lost, isn’t it.?” (Specialist palliative care focus group-Case 1).

Lack of expertise in end-of-life care was complicated by paramedics and technicians acknowledging a lack of in-service expertise for palliative care related advice. Furthermore, they emphasised that service pressures led to a lack of support on scene and pressure to get back on the road:

“…I rarely seek advice in service… because the service has its own focus of: “why is the crew still on scene after ninety minutes dealing with this end-of-life case?”, “we need them out dealing with something else…” (Paramedic and technician focus group-Case 1).

Different perspectives between professional groups provoked some tensions, precluding inter-professional working and shared professional decision-making. Community nurses and specialist palliative care staff expressed some criticism of what they perceived to be a superiority by ambulance crews, which they argued led to them not listening and taking advice as part of joint working and shared decision-making processes:

“…There seems to be: “we’re the emergency team so, what we say goes” and it doesn’t really work like that, and it’s not great for joined working and decision-making…” (Community nurse focus group-Case 1).

Specialist palliative care clinicians recognised by viewing things through different “lenses” ambulance crews often prioritised reporting aspects not judged to be a priority by specialist palliative care clinicians, like vital signs. Differences in ways of seeing made joint working and decision-making hard:

“…It’s like the thing where you have lots of people feeling different parts of an elephant and trying to describe it. They see it as very much as an A, B, C, D, and they’re telling you about a patient in A, B, C, D, and I’m saying to them: “what do they look like?”, “are they talking to you?”…” (Specialist palliative care focus group-Case 1).

##### Confusion over what is allowed regarding administration of anticipatory medicines

There was varied understanding and interpretation of which anticipatory medicines paramedics could and could not administer, including on the part of paramedics themselves. In the words of a specialist palliative care pharmacist:

“…So, the paramedics can only give what they’re allowed to give (laughing), and that’s often the difficulty… Sometimes we’ve had great paramedics who kind of listen and will, look at the authorisation chart [medicines authorisation and administration record] and go: “I can see what you’ve put in place here” and: “this is what we can give”, and whatever, but other times, it’s just been a bit of a block…” (Specialist palliative care focus group-Case 1).

Within the case (as in Cases 2 and 3) all paramedics were legally able to administer patients’ own anticipatory medicines (with the prescription acting as a patient specific direction, an instruction to administer a medicine to an individually named patient) and supported by the Trust’s medicines management policy. Many however referred to not being ‘allowed’ to administer these or needing to speak to a palliative care team first. Another available option was the use of morphine (carried on all ambulances) and authorised for use in the end-of-life context via Joint Royal Colleges Ambulance Liaison Committee (JRCALC) guidance. This was more widely recognised by paramedics as something they could do:

“…We are authorised at our Trust to give Morphine for end-of-life. We can’t give it from her anticipatory meds because it’s not something that we are allowed to do…” (Paramedic 3-Case1).

##### Limited access to patient records contributes to working in the dark

Limited access to electronic patient records meant paramedics were reliant on their own detective work (listening to family accounts and searching for paper-based documents and medicines). They were “working in the dark” and fortunate if able to contact a specialist palliative care service lead who could do the detective work on their behalf (because of their access to records) and ensure an informed decision about “*exactly what to do*”:

“…Sometimes things get escalated to the consultant on-call not necessarily because it’s the expertise of our, you know, consultant knowledge or anything that are needed, it’s because we have access to all systems. I’m an NHS consultant with an honorary contract to [hospice], and I also have access to the [record system], which is our computer record, for the other two hospices in our patch, plus the shared care record, and I can access the hospital records as well. So, sometimes, my role is literally as a detective to work out what’s going on so…” (Specialist palliative care focus group-Case 1).

### Case 2 – Anticipatory medicines carried by advanced paramedics

In this case, where advanced paramedics carried anticipatory medicines, they were an important source of remote support for on-scene paramedics and easily accessible 24/7 via an in-service hub. Advanced paramedics were often called by the attending crew for advice during the visit and this advice informed the final decision or recommendation of the crew. Nonetheless, data showed advanced paramedics rarely attended scene.

#### Patient and carer experience

##### Context and nature of callouts

Within this case most patients had some form of advance care planning related document in the home and connected with general practitioner and community nursing services for their palliative and end-of-life care needs; but this contact was more developed for some than others. Despite this, callouts to the ambulance service were made when symptoms were perceived to be out of control, usually in the out-of-hours period, in the knowledge that other services would not be contactable. Callouts were often connected to breathing difficulties (most patients recruited were living with chronic obstructive pulmonary disease) and families experienced panic reactions in response to what they were witnessing.

##### Patient preferences clearly stated

A notable aspect of these data were strong patient views on not wishing to go to hospital, or conversely for a minority, not wanting to die at home. For some their preferences were contrary to what the ambulance crew initially decided was the best course of action.

“…I couldn’t get up and they didn’t want to leave me like it. I didn’t have any strength to get up at all. But they’re— always asking me to go to hospital and I say: ‘I don’t want to. That’s why I’m with the hospice’…” (Patient-01-Case 2).

In some callouts the ambulance crew decision changed during the attendance as they liaised with others e.g. when the patient’s general practitioner confirmed that the patient’s wishes for non-conveyance, or they found advance care planning documents in the home:

“…Once, they’d [ambulance crew had] spoken to the GP surgery, they seemed a little bit more OK, they didn’t seem as concerned… I don’t know what they got from the doctor, but it seemed to reassure them what they were doing was OK… they needed someone to take that decision because they were adamant about the hospital really…” (Bereaved Carer-08-Case 2).

In most attendances, the crew deferred to a patient’s wishes which meant enabling the patient to remain at home, with onward referral to other services.

##### Limited access to patient records

Patient history was always elicited during attendance. This case did have the benefit of an electronic record system with a repository for advance care planning documents meaning crew could in theory be made aware of a patient’s advance care planning related documents via the system. But in all reported instances there was a focus on tracking down paper documents in the home, including DNACPR (Do Not Attempt CardioPulmonary Resuscitation) decisions or ReSPECT (Recommended Summary Plan for Emergency Care and Treatment) plans, to aid decision-making. This was reported as due to the documents not being in the online repository, not being trusted as contemporary or wanting to see original documents.

“…They [the ambulance crew] wanted to know if we had that document [DNACPR form], to them that was crucial…” (Bereaved Carer-08-Case 2).

Several patients and families commented clearly about the need for ambulance crew to have better access to patient records which would have avoided the need to repeat patient details or, assert wishes in already stressful situations:

“…It is a shame where, you know, in the height of all the anxiety and cuffuffle, and everything that’s going on, you have to go through a whole load of symptoms, all the medication, wait while it’s all written down, and I know there are probably reasons for this, but if only they could just bring up the information and it was there. I mean, it might be the previous two calls, where they were within a— I think, it was even on the same night, and yet the information they couldn’t access it…” (Bereaved Carer-09-Case 2).

##### Advanced paramedic practitioners are an important source of remote support

Advanced paramedic practitioners were often called by the attending crew for advice during the visit. These paramedics, unique to this case, carried three core anticipatory medicines (midazolam, cyclizine and hyoscine butylbromide) and were trained in administration. Advanced paramedic practitioner advice (both symptom management and medicines related) often informed the final decision or recommendation made by the on-scene crew. In only one instance did an advanced paramedic practitioner attend the scene, visiting at the request of the initial crew to assist with medicines administration in a presentation of urgent pain management.

##### Positive views of the ambulance service

Patients and families were very positive about their ambulance service experiences. Crew were described as very kind, calm, supportive, caring, professional and providing reassurance. There appeared to be a serious, unhurried consideration by crews of a patient’s and family’s wishes with careful assessment. In a minority of instances, patients and families reported less positive experiences, sometimes associated with more junior staff who were uncertain what they were doing.

“…The ambulance service… They’ve been absolutely great… I know that [specialist] palliative care is there if I need them, but I’ve had more contact, I think, with the ambulance people… they’re doing a wonderful job, and I’m very, very grateful…” (Patient-01-Case 2).

#### Healthcare professional perspectives

##### Proactive management to prevent callouts

Other groups of healthcare professionals, particularly community nurses and general practitioners, often via their primary care-based paramedic colleagues reported undertaking a significant amount of proactive work to identify and manage patients at end-of-life. In doing so, they were trying to prevent ambulance callouts. There was a suggestion that paramedics were more likely to be called to those patients who engage less with advance care planning and proactive care, or individuals not yet identified as being in the last year of life particularly those experiencing frailty.

“…We try and do everything in our power to avoid paramedic attendances for end-of-life patients. So, you know, we put ReSPECT forms in wherever we can, you know, all our Band 6’s and 7’s have ReSPECT training, and our experienced Band 5’s, we put forward for the ReSPECT Level 2 training so, if a paramedic does attend, we’ve got a good idea of patient wishes and they’ve got a good idea of patient wishes…” (Community nurse focus group-Case 2).

Despite the above, even where advance care planning took place, associated documents were often reported by paramedics as incomplete, out-of-date, could not be found in the home, or overtaken by events and a family understandably panicked by the circumstances of the callout.

##### Depleted community services lead to callouts

There was a commonly held view that paramedics are increasingly called to patients at end-of-life because of the depletion of other services, particularly community nursing and loss of senior experience within community nursing services, and failure of out-of-hours services. There was also geographical variation in in- and out-of-hours services across this ambulance service region. There was a good deal of sympathy for paramedics facing these situations:

“….You’ve got your district nurses, your acute response team, your frailty service, paramedics, the hospices. So, between those to hold people in the community should be more than possible but it depends on the funding or actual members in teams, and it always comes back to the fact that, you know, sometimes your teams and community teams do not have enough staffing to hold people in the community, and I find that very, very sad because we’re going round and round in circles…” (Specialist palliative care focus group-Case 2).

##### Variable knowledge supported by remote advice services

A view shared by all professional groups was one of variability in paramedics’ knowledge and skills in being able to manage patients at end-of-life, despite the end-of-life care specific in-service training that was a feature of the case.

“…How much knowledge do the paramedics have with regards to palliative patients? And, you know, if symptoms do arise, are all of them aware of how to manage those symptoms? Yeah, because there’s been a few incidents… Some have been fantastic and know exactly what to do, but others, yeah, aren’t quite sure what to do in that situation…”. (Community nurse focus group-Case 2)

Experience was deemed important and more prevalent than any kind of structured pre-registration or post-registration training. It was evident that support was often sought from the in-service advanced paramedic practitioner hub or from 24/7 specialist palliative care lines where available.

##### Limited access to records is a barrier to good care

There was wide recognition from most healthcare professional groups that paramedics’ lack of access to shared records was a significant barrier in providing good care. It also sometimes led to avoidable conveyance when the paramedic was unaware of the patient’s end-of-life history or other complexities surrounding their care and underlying conditions.

“…We need to improve the information that we give to the ambulance Trust and make that a little bit more thorough, and helpful for them. Maybe what we could think about is giving them people on our red list [Gold Standards Framework], to let them know who’s on our radar. I wonder if that kind of information is able to be shared [with the ambulance Service] because essentially, we’re all part of the same confidential sort of environment…” (General practitioner-Case 2).

##### Limited inter-professional understanding and integration

There was some evidence from community nurses and specialist palliative care nurses that paramedics might lack understanding of nursing services and/or not fully respect their decisions and advice to convey or not to convey. Data from all groups indicated some lack of understanding of each other’s services and certainly lack of integration of services in terms of ability to contact each other and share records.

“…We don’t get any learning from our involvement with them (ambulance service) particularly, and that might be nice …it would be useful to see from their perspective, what would be more helpful for them, what they’re struggling with…” (Specialist palliative care focus group-Case 2).

##### Blocks to providing and administering anticipatory medicines

With regards to anticipatory medicines, there was a prevailing view from paramedics that because they had not been specifically trained in their administration, they would not be confident enough to administer them even if they were available in the patient’s home. Problems with incomplete or missing medicines authorisation and administration records also seemed to be a block to timely administration of medicines by paramedics. There were no illustrative examples in the healthcare professional data of advanced paramedic practitioners visiting the scene to give these medicines (although there was one episode in the patient data). Most other healthcare professionals, particularly general practitioners and community nurses, were unaware that core anticipatory medicines were carried by these advanced paramedics.

### Case 3 – In-service specialist palliative care team

Within this case, the value of “palliative care transfer ambulances”, acting as a separately commissioned transfer service at imminent end-of-life (but not responding to 999 calls), was highlighted. These vehicles were highly responsive, and crews recognised as going “above and beyond”. Patients and families were often asked what would make a difference to the journey, and as a result they often travelled via meaningful locations. Community-based healthcare professionals and specialist palliative care clinicians provided accounts of touching and exceptional service.

#### Patient and carer experience

##### Context and nature of callouts

Patients and carers in this case provided data on callouts for the most diverse range of reasons across the cases [dementia, falls, chest infection, cancer, heart disease] which may have been related to the case’s geographical location in the North of England with long-standing health inequalities and industrial past [35]. Patients acknowledged they had some form of advance care planning document in place: either a DNACPR only or a DNACPR plus emergency health care plan or advance care plan. However, in all callouts patients and carers were asked to provide ambulance crews with paper copies of their advance care planning documents, despite the case (as in Case 2) having an electronic record system with a repository for advance care planning documents. In practice this did not appear to be making a difference to frontline paramedics.

“…I just don’t think [advance care planning] records are shared at all between anybody… it was down to me to keep the care provider [ambulance crew] updated which was fine. The district [community] nurses were kept updated because they said they use the same system so they could see everything that was going on in hospital. So, they were the only ones who were kind of kept in the loop really…” (Carer-02-Case 3).

In some instances, misunderstanding of advance care planning related documents resulted in calls to the ambulance service (where domiciliary care workers rang 999 counter to preferences documented in the advance care plan) and at least one hospital conveyance.

#### Healthcare professional perspectives

##### Lack of identification of individuals as being in the last year of life

There was consensus amongst healthcare professionals that primary care, community nursing and specialist palliative care services were working hard to provide skilled, pre-emptive care that enabled patients to avoid calling ambulance services. However, healthcare professionals recognised that this could become unavoidable especially out-of-hours with acute and reversible emergencies, patient panic (especially when living alone) and family members, and for those who had not previously been identified as being in the last year of life, particularly amongst frail older populations.

Poor identification of individuals being in the last year of life led to a common experience expressed amongst paramedics and ambulance technicians of not knowing that a callout was for an individual in their last year of life before arrival.

Demographic complexities of non-malignant, long-term conditions e.g. dementia, frailty, falls, heart disease in comparison to more predictable condition trajectories such as cancer, with greater challenges in identifying the last year of life, may have raised the likelihood and need for ambulance callouts:

“…If patients haven’t been obviously identified as end-of-life, and that happens more in our frail elderly patients, rather than our cancer patients – those [frail older] patients tend to not get escalated to the out of hours palliative care, they’re the ones that end up being the ones picked-up by the paramedics…” (General practitioner1-Case3).

##### Late referrals to community nursing services and depleted provision leads to callouts

In line with the complexities of recognising non-cancerous end-of-life trajectories, community nurses discussed late referrals to their services (during the last few months of life). Furthermore, issues of late referral appeared compounded by lack of capacity within community nursing services which could lead to ambulance service callouts:

“…They might not be referred into our service until they’ve only got three months left to live… there might be a section of— that that we’re just not capturing because we don’t know about the patient or we’re not seeing them regularly enough…” (Community nurse focus group-Case 3).

Despite recognition of depleted community nursing service provision leading to callouts, there was also recognition that paramedics were lucky to have access to 24/7 community nursing services (via a single point of access).

##### Variable paramedic skillsets

Specialist palliative care clinicians referred to variable experiences for patients due to a lack of clinical knowledge amongst paramedics and difficulties distinguishing what might be reversible:

“…Sometimes we’ve seen excellent examples of, yes, you know, absolutely this patient really does need to stay home and, you know, palliative care and follow-up out of hours GP service or their own GP would be really appropriate, but then there would be other situations where perhaps the judgement of the paramedic wasn’t a 100% …. You know, there’s lots of things that can masquerade as a deterioration in a patient that can masquerade as end-of-life reversible causes…” (Specialist palliative care focus group-Case 3).

Community nurses perceived the paramedic skillset as most aligned with acute end-of-life care scenarios which need hospital treatment. They spoke of paramedics doing “*well in that area*”. Specialist community based palliative care clinicians (not those in the in-service specialist team) spoke of being called by paramedics on scene for support, guidance and signposting. They referred to being asked whether patients could be admitted to the hospice as an alternative to conveying them to hospital, and that queries often related to advance care planning.

Underpinning variable skillsets was a lack of palliative and end-of-life care education and training. Despite the in-service specialist team and provision of end-of-life care specific in-service training (both key features of the case) training did not appear to be sufficiently frequent, effectively sign-posted or accessible. It was criticised as reliant on paramedics seeking it out themselves, via external providers and in their own time:

“…We haven’t actually received a lot of training, and the training that is offered to us is— normally, it’s external service CPD [continuing professional development] often provided by [organisation], and that has to be done in your own free time so, we don’t really get much support when it comes to palliative care, end-of-life care. A lot of it is very much learn it as you go …” (Case 3, Paramedic 3).

##### Absent or variable quality of advance care planning documents

In contrast to patient and carer data, there was consensus amongst healthcare professionals that advance care planning processes had often not been undertaken so documents were completely absent, or where they did exist, not always fit for purpose in directing a paramedic during a 999 callout. Absent or poorly completed advance care planning documents left ambulance crews without clear guidance in an emergency:

“…We also need to focus on the quality of documents left with the patients. Some emergency healthcare plans are very straightforward, especially those written by palliative care specialists, Macmillan nurses, those are very good. Some are absolutely rubbish, sorry for saying it, but information a patient is for full escalation on the emergency healthcare plan, it’s not giving you much support…” (Paramedic and ambulance technician focus group-Case3).

Healthcare professionals all referred to the challenges of having to locate paper copies in the home, where *“….half the time the patients don’t know where it is…” (Specialist palliative care focus group-Case 3).*

This ambulance service reported they hold about half of all DNACPRs for their service population (due to engagement work done by the in-service specialist palliative care team with primary, community and secondary care providers to encourage providers to share these documents with the ambulance service). However, the system of flagging DNACPRs at the point of call and then making these accessible electronically to paramedics appeared fragmented and flawed:

“…So, what happens with our [electronic record] system is obviously, there’s— put a flag on it, and then the patient will have a patient file. Unfortunately, road staff aren’t— them files aren’t visible. There’ll be the flag on and then we need the dispatch clinician to actually be able to access some of them files. So, for all, there’ll be a flag on saying there’s a DNACPR, the best thing it does for us is to have that in your head to know when you go in actually: ‘we know there’s one, it’s on file, but where is it?’ kind of thing because: ‘is it in date?’, ‘when was that note put on.?’ (Paramedic and ambulance technician focus group-Case3).

##### Confusion over providing and administering anticipatory medicines

All paramedics could administer a patient’s own anticipatory medicines, and specialist paramedics carried midazolam. However, there were many comments from paramedics about confusion on what they can do (as well as a lack of awareness from other healthcare professionals including specialist palliative care clinicians and general practitioners on what was possible), with relative low levels of confidence and fears.

“…I think, as clinicians we’re getting really much better at it but I still think there’s the bit of a hesitation that: ‘oh, my God, what if I’ve made the wrong decision, the doctor hasn’t put i’s on the paperwork in place’, but I’m quite happy once I’ve spoke to a GP, once I’ve recognised it, once I’m happy with it, that, you know, I’ll absolutely start because the care of the patient, you know, starts with the pain relief and the less of the agitation, and normally that’s the reason the family have called because they look in pain, they appear agitated…” (Paramedic and ambulance technician focus group-Case3).

One of the general practitioners interviewed stated:

“…I’ve never had a (ambulance service) colleague administer any end-of-life meds to any of my patients. Whether that’s because --- the need hasn’t arisen rather than they can’t do it…” (General practitioner2-Case3).

Specialist palliative care clinicians reflected on the need for training to administer these types of sub-cutaneous medications, but they too were uncertain whether paramedics were able to administer patient’s own anticipatory medicines:

“…I recently taught on a Master’s [level] class for paramedics about symptom control in palliative care, and most of the questions I was being asked were about sub-cut injections, sub-cut infusions, getting more training and how to give those medications and just giving them a bit more knowledge and information about symptom control at the end-of-life. I’m not sure if paramedics can— if someone’s got anticipatories in the house whether they can administer that…” (Specialist palliative care focus group-Case 3).

This lack of awareness, on whether these medicines could be administered, was also discussed by paramedics themselves:

“…A lot of paramedics don’t realise they can give those drugs, because they’re not comfortable with these subcutaneous injections…” (Paramedic 2, Case 3).

A lack of associated training contributed to this confusion and apparent reluctance to administer these medicines. There was more confidence regarding administering morphine, carried on all vehicles and authorised for use by JRCALC guidance:

“…I’ve never had to [administer patients’ own anticipatory medicines]… Obviously Morphine, that’s not an issue. I’ve never actually had to administer any of the other medications so, if it was a case of, I need to do that now, I would probably— well, we’ve got our [clinical advice hub], I’d obviously check that there and, probably get some advice from a manager and just sort of clarify… the dosages and so forth…” (Paramedic 1,Case 3).

There were however some inaccurate fears expressed that use would hasten death.

##### Dedicated end-of-life care transfer vehicles facilitate death in preferred place

Community nursing teams and specialist palliative care clinicians alike spoke of the “*huge difference*” the provision of palliative care ambulances made (commissioned via the ambulance service but externally provided). They spoke of their awareness of the service, its value in enabling patients to travel to their preferred place of death and timeliness of provision (within an hour of referral during service hours).

“…We’re quite lucky in [this] ambulance service because we’ve got a service that provides an end-of-life ambulance with a one-hour rapid response. I don’t know whether other areas have it, but it’s a very, very useful service that we find whether that’s patients coming out of hospital to home or whether that’s us in the community admitting somebody to the hospice, and it just helps with that seamless care and it’s better for the patient…”(Community nurse focus group-Case 3).

They recognised the crews of these vehicles as going “above and beyond” in the care provided, and provided touching examples of crews asking patients for requests for their final journeys and ensuring these were met e.g. travelling via locations with personal meaning.

“…What they do, and they do really well is having that empathy and understanding of palliative care needs when they’re transferring patients. I think, it’s always really reassuring as a nurse that if you think you’re handing over the care to somebody who genuinely cares as well, and I have to say, you know, we do come across some fantastic ambulance staff…” (Specialist palliative care focus group-Case 3).

### Cross-case analysis

Across the cases, positive views of paramedic care and ambulance service provision were provided by patients and carers. Similarly, healthcare professionals spoke of their empathetic attitudes towards paramedic colleagues and the situations they often faced, with limited supporting resources (records, medicines and advice). Despite this, data revealed tensions between professional groups because of different perspectives and limited levels of inter-disciplinary and inter-organisational collaboration.

Despite sampling cases (2 and 3) with service delivery characteristics potentially supportive of paramedic delivered end-of-life care, several pervasive challenges were consistent across all three cases, negating the impact of these facilitatory features (anticipatory medicines carried by advanced paramedics in Case 2 and in-service specialist palliative care team in Case 3).

#### Primary and community care service shortages

Shortages of staff and services in the wider health service system often drive paramedic attendance. Paramedics were frequently called to manage situations resulting from breakdowns elsewhere in the healthcare system. Patients and carers reported inconsistent support from core generalist services, such as general practitioners and community nursing teams, as well as specialist palliative care services (including hospices).

#### Challenges in interdisciplinary collaboration

Limited access to patient healthcare records was evident across all cases, with paramedics operating with very limited access to electronic records. Paramedic record access was usually confined to access to ambulance service records if a crew had been out previously. Therefore, they had to rely on their own detective work, family insights, or searching homes for documentation. Despite two of the cases using their service’s record system as a repository for advance care planning documents, this did not seem to make a difference to paramedics in practice.

Commonplace were legacy views of paramedic scope of practice and ambulance service provision. Misunderstanding across the healthcare system regarding the contemporary scope of paramedic practice and ability to facilitate complex home-based care (as opposed to hospital conveyance), undermined inter-disciplinary collaboration and limited shared professional decision-making, essential for best practice at end-of-life.

#### Limited education and training in end-of-life care

All healthcare professional groups referred to limited education and training for paramedics regarding palliative and end-of-life care. Although two of the cases reported to provide end-of-life care specific in-service training, participants did not appear to be aware of this provision or have been the beneficiaries of it. In the main paramedics referred to a complete absence of ambulance service education provision and needing to access training themselves through external providers, in their own time.

Limited education and training led to variable skillsets and a reliance on gaining knowledge and expertise entirely through experiential learning. Limited education and training enabled fears and/or uncertainty regarding palliative and end-of-life care to accumulate. Concerns included missing potentially reversible or treatable issues, distinguishing the dying phase, administering subcutaneous medicines, knowing and administering the correct stat or prn (as required) dose for an individual, and concerns over respiratory suppression with morphine.

#### Challenges with medicines access and administration

Paramedics in all cases faced barriers to accessing and administering anticipatory medicines, which undermined their ability to manage symptoms effectively. Where patients’ own anticipatory medicines were available in the home there was confusion over what was allowed by their ambulance service, this combined with a lack of training on these medicines, led to widespread uncertainty and reluctance to administer patients’ own anticipatory medicines. Advanced paramedics able to administer independent supplies of these medicines were useful sources of support for crew at scene but were too thinly spread to directly administer medicines in patients’ homes and avoid the delays incurred by referral to other prescribers. There was some evidence of greater willingness to administer morphine - universally accessible by all paramedics.

## Discussion

This study is the first to report on paramedic practice at end-of-life using the lens of service models. It was conducted across England utilising in-depth case-studies, drawing on mixed method data collection from over 100 (*n* = 101) participants including the patient and carer voice. Through this empirical work we have demonstrated that paramedic practice for individuals in their last year of life varies across ambulance services, and within services. This variation, in part, can be accounted for by a lack of knowledge, skills and confidence (hampered by fear and uncertainty) resulting from insufficient provision of end-of-life specific education and training. Furthermore, practice was impeded by limited access to essential resources - be these records, advice and/or medicines.

To address this widespread variation solutions are needed that adopt a system-wide approach to ensure paramedics are well-connected to the wider healthcare team [36]. Paramedics must be supported via improved access to timely advice and referral pathways with generalist and specialist palliative care providers to enable effective shared decision-making, underpinned by access to digital shared patient record systems. Our findings indicate that palliative and end-of-life care training should be provided for all frontline paramedics. Addressing knowledge gaps in end-of-life care, via the delivery of accessible training packages that include simulation methods, is essential to help paramedics practice without fear and uncertainty. Other studies (UK and Australia) have examined the effectiveness of various modes of end-of-life care training for ambulance clinicians [37–42]. Ambulance clinicians report end-of-life care training improves confidence, feeling more able to act in the best interests of patients, better able to communicate and more confident to administer necessary medicines [37–42]. Effective implementation of such training would go a long way to address the foremost underpinning system-level issue (insufficient provision of end-of-life specific education and training) underpinning existing practice variation within and across services.

An example of a system-wide approach to ensure paramedics are well-connected to the wider healthcare team has been implemented by the Scottish Ambulance Service [43]. It has embedded palliative education alongside decision support via access to senior clinical advice and ~ 30 alternative referral pathways e.g. community palliative teams, out-of-hours lines and hospice in-reach. The service has trained over 4000 staff, reducing hospital conveyances by 5% or 1600 fewer conveyances and saved an estimated £3.6–5.6 million when compared to 2022 and 2023 [43]. This service model could be cost-effective in reinforcing the ambulance service’s critical role in enhancing end-of-life care more widely [43]. But it requires palliative and end-of-life care education and training to reach all frontline paramedics supported by accessible, best practice advice and referral pathways whilst on scene. It is the combination of education/training with access to quality advice/decision-support and referral pathways that appears critical in successfully negating the system-level issues occurring across services and better supporting paramedics to reduce practice variation.

Our case studies illustrate the importance of access to and ability to confidently administer anticipatory medicines by frontline paramedics. The findings suggest that there is currently reliance on a limited number of advanced paramedics in a service delivery system to support this. Both patient record and interview data showed these advanced paramedics rarely attended scene. This may reflect relative low numbers of advanced paramedics in relation to the geographical footprint of a service – reducing the ability to be in the right place at the right time to make attendance feasible. Such an approach may not be realistic nor sustainable, given the rising population of people likely to need palliative care symptom management at home [20, 21]. To support a more distributed model with all frontline paramedics able to administer patients’ own anticipatory medicines, there needs to be clear communication both within and across service organisations nationally regarding medicines management policies i.e. what is permissible. It is essential this is combined with training to instil confidence, particularly regarding calculating breakthrough doses, drawing up medicines from ampules, and the sub-cutaneous route of administration that is different to what paramedics might administer in other areas of urgent and emergency care. Our national survey reported elsewhere [22] also indicates that prescribers and healthcare teams need to make correctly completed medicines authorisation and administration record (MAAR) charts more consistently available to support administration of these medicines by frontline paramedics in patients’ homes. This again highlights the inter-connectedness of paramedic practice with the wider healthcare team and the system-wide approach needed to support a strengthening of paramedics’ practice. The availability of anticipatory medicines and MAAR charts in people’s homes, has also been found to be a problem in community nursing contexts [25, 44–46], indicating cross-disciplinary inter-connectedness underpins effective medicines administration in all end-of-life contexts, but the consequences of this are heightened during paramedic attendance where frontline paramedics are more isolated from other healthcare professional / palliative care team members.

Limited research suggests that where anticipatory medicines are carried on emergency ambulances with paramedics delivered to administer them, there are no adverse events and cost-savings arise due to avoidance of hospital conveyance [47–49]. The potential value of such an approach is in the availability of anticipatory medicines to all paramedics within an ambulance service, rather than a dependence on availability of patients’ own medicines whilst on scene. This is important in providing timely symptom relief for individuals, reducing distress for patients and their families, and impacting family members bereavement outcomes where an individual goes on to die [48]. However, such an approach requires a comprehensive training package to be implemented alongside the availability of the medicines on ambulances [48].

Our research has endorsed the clear value of palliative care ambulances operating outside, but in addition to, usual emergency provision. One other UK study of specialist transport services being used for patients approaching death, also found that dedicated palliative care ambulances can be successfully used to facilitate transfer of individuals to their preferred place of death [50]. Such services have historically been provided by charities, and where palliative care ambulance provision is to be extended it may require ambulance services to work collaboratively with such providers.

### Limitations of the study

Ambulance service models vary by region and nation across the UK, and this variety is compounded globally where there are different models of service and paramedic practice. This study will have most relevance internationally to countries where the nature and scope of paramedic practice is aligned with UK practice. This is most likely for those countries practicing an Anglo-American emergency medical service model (paramedic-led) namely the Republic of Ireland, USA, Canada, Australia, most of the Middle East and South Africa, as opposed to the Franco-German model (physician-led) pertaining to France, Germany, Italy and Spain. Despite these differences we believe the underpinning key issues surfaced across the service models studied have relevance outside England and internationally.

Patient recruitment was lower than targeted in one of the case studies (Case 3), due to late referrals to specialist palliative care via the participating hospices (where individuals were near to death) and significant service pressures [51–53]. Although patients were identified as eligible, the focus of clinical staff on service delivery meant those eligible were not often approached. For example, *n* = 191 patients were screened in one of the two hospice sites, *n* = 50 of whom were deemed eligible but only one (*n* = 1) invited to participate. Nevertheless, information power was gained within the cases via robust purposeful sampling, in-depth expertly conducted interviews and focus groups, and recruiting to target overall, whilst utilising carer recruitment to supplement patient voices.

## Conclusions

The study has provided an in-depth exploration of paramedic practice at end-of-life via case studies of ambulance service models. The cases highlighted widespread variation in practice impacting experiences for individuals in their last year of life. In the main paramedics are working without the benefit of in-service specific palliative and end-of-life care training, universal round the clock access to specialist palliative care advice, or access to shared electronic patient healthcare records, and at times without access to patients’ own anticipatory medicines. This complex context allows widespread variation to exist, with practice dominated by learning on the job and hampered by limited access to resources. Access to advanced paramedics who carry anticipatory medicines (utilised for accessible 24/7 in-service advice rather than on scene attendance) and palliative care ambulances facilitated care provision and patient outcomes (death in preferred place). Nonetheless, if paramedics are to be in the best position to address patients’ needs more attention is required to promote access to in-service palliative and end-of-life care training and healthcare records (including advance care planning documents) via ambulance service systems.

## Supplementary Information

Supplementary Material 1.

Supplementary Material 2.

Supplementary Material 3.

## Data Availability

The datasets used and analysed during the study are available from the corresponding author on reasonable request following required ethical approvals.

## Abbreviations

DNACPR: Do Not Attempt Cardiopulmonary Resuscitation
GP: General Practitioner
JRCALC guidance: Joint Royal Colleges Ambulance Liaison Committee guidance
MAAR charts: Medicines Authorisation and Administration Record charts
NHS: National Health Service
PGD: Patient Group Direction
ReSPECT: Recommended Summary Plan for Emergency Care and Treatment
UK: United Kingdom
