# Supplementary material for: Investigating the impact of ambulance service models on paramedic delivery of end-of-life care in England: an exploratory study using case study design
Source: BMC Palliat Care. 2026 Aug 12;25:322. doi: 10.1186/s12904-026-02283-x (PMC13692981; doi:10.1186/s12904-026-02283-x)
Supplement: Supplementary file 1 [file 12904_2026_2283_MOESM1_ESM.docx]

**Study Title:** **Paramedic delivery of end-of-life care (ParAid Study): a mixed methods evaluation of service provision and professional practice**

ERGO 90972; IRAS 340051 - V1– FEB 2024

**-------------------------------------------------------**

**Paramedics and Ambulance Technicians – Focus Group Guide**

**Purpose**: to understand your experiences of delivering care to those in the last year of life, influencing factors that affect the context of delivery and how service delivery features specific to your ambulance service influence ability to deliver care. We would also like to explore your views on barriers and facilitating factors both in and out-of-hours.

- How do you see your roles in attending patients at end-of-life?
- Could you share general / common experiences of attending patients in this context?
- Could the group reflect and share on recent end of life callouts?
  - What went well/not so well,
  - What influenced your actions on scene and outcomes?
  - What aspects of the service delivery in your Trust influenced your actions/care?
  - What aspects of surrounding/local service provision influenced your care/actions? e.g. specialist palliative care team, community nurses and GPs
- What might be done differently in the future – how service changes might bring about improvements for staff, and changes to improve outcomes for patients?
- Is there anything that we haven’t discussed that you would like to tell us about?

**-------------------------------------------------------**

**Community Nurses / Specialist Palliative Care Clinicians – Focus Group Guide**

**Purpose**: to explore your experiences of paramedic / ambulance service delivered end of life care, and the factors that influence this care both in and out of hours. We are also interested in the interplay between your own services and the ambulance service, and vice versa.

- Could you share your experiences of paramedic / ambulance service delivered end of life care?
- What factors influence the nature of this care?
  - Probe: factors in and out of hours
  - Probe: shared record systems, advance care planning conversations and documentation, access and availability of patient’s own JIC medicines
- Could the group reflect and share on recent 999 callouts/ambulance attendance for patients on your caseloads at end of life?
  - Why were the calls made?
  - Are you aware of what happened on scene?
  - What were the outcomes of attendance for your patients?
  - What aspects of your own service influenced the need for the call/ambulance attendance and what happened as an outcome of the attendance?
  - Were there impacts on your service as a result of the call/ambulance attendance? Probe:
    - Were you contacted on scene/involved in on scene referrals, what shared decision making was undertaken?
    - When and how were you informed of the call/attendance?
  - If we consider the nature of paramedic delivered end of life care (the specifics of the service provision) in your region are you aware of what impact these have?
    - Probes to be tailored to the case e.g. availability of advanced paramedics that carry JIC medicines…
- What might have been done differently – how service changes might bring about improvements for staff, and changes to improve outcomes for patients?
- Is there anything that we haven’t discussed that you would like to tell us about?

**-------------------------------------------------------**

**General Practitioners – Interview Guide**

**Purpose:** to explore your experiences of paramedic / ambulance service delivered end of life care, and the factors that influence this care both in and out of hours. We are also interested in the interplay between your own GP service and the regional ambulance service, and vice versa.

- Could you share your experiences of paramedic / ambulance service delivered end of life care?
- What factors influence the nature of this care?
  - Probe: factors in and out of hours
  - Probe: Identification of patients as at end of life (via palliative care registers), shared record systems, advance care planning conversations and documentation, access and availability of patient’s own JIC medicines
- Could you share recent 999 callouts/ambulance attendance for patients on your caseload at end of life?
  - Why were the calls made?
  - Are you aware of what happened on scene?
  - What were the outcomes of attendance for your patients?
  - What aspects of your own service influenced the need for the call/ambulance attendance and what happened as an outcome of the attendance?
  - Were there impacts on your service as a result of the call/ambulance attendance? Probe:
    - Were you contacted on scene/involved in on scene referrals, what shared decision making was undertaken?
    - When and how were you informed of the call/attendance?
  - If we consider the nature of paramedic delivered end of life care (the specifics of the service provision) in your region are you aware of what impact these have?
    - Probes to be tailored to the case e.g. availability of advanced paramedics that carry JIC medicines…
- What might have been done differently – how service changes might bring about improvements for staff, and changes to improve outcomes for patients?
- Is there anything that you haven’t already discussed that you would like to add?

**-------------------------------------------------------**

**Patient and Carer – Interview Guide**

**Purpose:** we would like to explore your experiences of paramedic/ambulance delivered care during most recent 999 call/s.

Thinking about the most recent 999 calls and attendance, could we explore:

**The call**

- Who made the 999 call? What was the reason for the call?

**Attendance and assessment**

- What did the crew think the reason for attendance was?
- What happened on arrival - how did they assess what was going on for you and what was important for you?
- What did you need to explain / tell them about?
- What could they access on scene – were there any documents at home? Or other documents they needed from somewhere else?

**Treatment and Care**

- What happened next?
- Did they give medicines (were these from home supplies)? What effect did they have?
- Do you know which other services they spoke with?
- Were you moved somewhere else, or referred onto another service?

**Support**

- Who supported you at the time?
- How did the ambulance staff communicate with you? Did you feel you were involved in decisions? How did you feel supported generally?

**Last questions**

- Now that you are looking back on the experience have your views on it changed in any way? Was anything missing? Could anything have been improved?
- Is there anything else you would like to tell me about the experience that we have not already covered?
