# Supplementary material for: Investigating the impact of ambulance service models on paramedic delivery of end-of-life care in England: an exploratory study using case study design
Source: BMC Palliat Care. 2026 Aug 12;25:322. doi: 10.1186/s12904-026-02283-x (PMC13692981; doi:10.1186/s12904-026-02283-x)
Supplement: Supplementary file 2 [file 12904_2026_2283_MOESM2_ESM.docx]

**The Vignettes**

| VIGNETTE 1 | Call to a 58-year-old woman diagnosed 2 years ago with glioblastoma (brain cancer). In the last few weeks her disease has significantly progressed and her condition has deteriorated.  She now has:   - a care package (visits three times a day) - anticipatory (just in case) medicines - a DNACPR form - she has been referred to the community specialist palliative care team.   Her husband and 2 daughters provide support at home and are aware that her prognosis is limited. She previously stated that she wished to die at home.  At 03:00 she has become difficult to rouse, and her noisy and irregular breathing is upsetting the family, so they have called 999. |
| --- | --- |
| VIGNETTE 2 | Call to a 66-year-old gentleman with metastatic prostate cancer and extensive bone secondaries. He lives with his partner in a second storey flat. His partner called 999 when he came home from shopping and found him on the floor, unable to get up.  On arrival you find he has been incontinent of urine and is complaining of radiating lower back pain. He does not want to go to hospital, is requesting pain killers and to be moved into bed. |
| VIGNETTE 3 | In-hours call to a gentleman in his 80s with end-stage COPD. He was discharged from hospital three days ago following treatment for a chest infection. He was sent home with antibiotics and anticipatory (just in case) medicines. He’s reluctant to go back to hospital.  His daughter called an ambulance due to him refusing his oxygen and medicines.  On arrival you find him breathless and anxious. His daughter states there is “no paperwork” for the just in case medicines, and she seems distressed. |
