# Supplementary material for: Investigating the impact of ambulance service models on paramedic delivery of end-of-life care in England: an exploratory study using case study design
Source: BMC Palliat Care. 2026 Aug 12;25:322. doi: 10.1186/s12904-026-02283-x (PMC13692981; doi:10.1186/s12904-026-02283-x)
Supplement: Supplementary file 3 [file 12904_2026_2283_MOESM3_ESM.docx]

**Participant characteristics by case**

|  |  | **Sample group** | **Main diagnosis/**  **Relationship** | **Sex** | | **Age** | **Ethnicity** | |
| --- | --- | --- | --- | --- | --- | --- | --- | --- |
| **CASE 1** | PATIENTS & CARERS | Patient | Melanoma | M | | 61-70 | White/British | |
|  |  | Carer | Wife | F | | 61-70 | White/British | |
|  |  | Patient | Lung cancer | M | | 71-80 | White/British | |
|  |  | Carer | Wife | F | | 71-80 | White/British | |
|  |  | Patient | Heart disease | M | | 81-90 | Asian/Asian British | |
|  |  | Patient | Oesophageal cancer | F | | 51-60 | White/British | |
|  |  | Patient | Bladder cancer | M | | 81-90 | White/British | |
|  |  | Patient | Melanoma | M | | 71-80 | White/British | |
|  |  | Bereaved Carer | Daughter-in-law | F | | 61-70 | White/British | |
|  |  | Bereaved Carer | Daughter | F | | 41-50 | White/British | |
|  |  | **Sample group** | **Years since qualification** | **Sex** | | **Age** | **Ethnicity** | |
|  | HEALTHCARE PROFESSIONALS | General practitioners | 6 | F | | 41-50 | Asian/Asian British | |
|  |  |  | 26 | M | | 51-60 | Black/African/Caribbean/ Black British | |
|  |  | Specialist palliative care clinicians | 14 | F | | 51-60 | White/British | |
|  |  |  | 19 | F | | 41-50 | White/British | |
|  |  |  | 24 | F | | 41-50 | White/British | |
|  |  |  | 29 | F | | 51-60 | White/British | |
|  |  | Community nurses | 14 | F | | 31-40 | White/British | |
|  |  |  | 15 | F | | 41-50 | White/British | |
|  |  |  | 19 | F | | 41-50 | White/British | |
|  |  |  | 21 | F | | 41-50 | White/British | |
|  |  |  | 22 | F | | 41-50 | White/British | |
|  |  |  | 28 | F | | 51-60 | White/British | |
|  |  | Paramedics and ambulance technicians | 3 | F | | 18-30 | White/British | |
|  |  |  | 2 | F | | 18-30 | White/British | |
|  |  |  | 7 | M | | 41-50 | White/British | |
|  |  |  | 10 | F | | 31-40 | White/British | |
|  |  |  | 10 | M | | 41-50 | White/British | |
|  |  |  | 11 | M | | 31-40 | White/British | |
|  |  | Paramedics | 2 | F | | 31-40 | White/British | |
|  |  |  | 7 | F | | 41-50 | White/British | |
|  |  |  | 7 | M | | 51-60 | White/British | |
|  |  |  | 8 | M | | 31-40 | White/British | |
|  |  |  | 20 | F | | 41-50 | White/British | |
|  |  |  | 24 | M | | 51-60 | White/British | |
|  |  | **Sample group** | **Main diagnosis/**  **Relationship** | **Sex** | | **Age** | **Ethnicity** | |
| **CASE 2** | PATIENTS AND CARERS | Patient | Leukaemia | M | | 71-80 | White/British | |
|  |  | Carer | Wife | F | | 61-70 | White/British | |
|  |  | Patient | COPD | M | | 71-80 | White/British | |
|  |  | Carer | Wife | F | | 71-80 | White/British | |
|  |  | Patient  Carer | COPD  Partner | M  F | | 61-70  51-60 | White/British  White/British | |
|  |  | Patient | COPD | F | | 71-80 | White/British | |
|  |  | Bereaved Carer | Wife | F | | 71-80 | White/British | |
|  |  | Bereaved Carer | Wife | F | | 61-70 | White/British | |
|  |  | Bereaved Carer | Daughter | F | | 51-60 | White/British | |
|  |  | Bereaved Carer | Daughter | F | | 51-60 | White/British | |
|  |  | Bereaved Carer | Wife | F | | 41-50 | White/British | |
|  |  | Bereaved Carer | Son | M | | 18-30 | White/British | |
|  |  | **Sample group** | **Years since qualification** | **Sex** | | **Age** | **Ethnicity** | |
|  | HEALTHCARE PROFESSIONALS | General practitioners | 10 | M | | 31-40 | White/British | |
|  |  |  | 14 | F | | 51-60 | White/British | |
|  |  | Specialist palliative care clinicians | 6 | F | | 51-60 | White/British | |
|  |  |  | 18 | F | | 31-40 | White/British | |
|  |  |  | 32 | F | | 51-60 | White/British | |
|  |  |  | 38 | F | | 61-70 | White/British | |
|  |  |  | Optional demographic data not provided | | | | | |
|  |  | Community nurses | 1 | F | | 51-60 | White/British | |
|  |  |  | 4 | F | | 18-30 | White/British | |
|  |  |  | 4 | F | | 18-30 | White/British | |
|  |  |  | 9 | F | | 31-40 | White/British | |
|  |  |  | Optional demographic data not provided | | | | | |
|  |  | Paramedics and ambulance technicians | 0 | F | | 31-40 | White/British | |
|  |  |  | 0 | F | | 31-40 | White/British | |
|  |  |  | 1 | F | | 41-50 | White/British | |
|  |  |  | 1 | F | | 18-30 | White/British | |
|  |  |  | 1 | M | | 18-30 | White/British | |
|  |  |  | 1 | F | | 31-40 | White/British | |
|  |  |  | 1 | M | | 31-40 | White/British | |
|  |  |  | 2 | F | | 18-30 | White/British | |
|  |  |  | 4 | M | | 31-40 | White/British | |
|  |  |  | 4 | F | | 41-50 | White/British | |
|  |  | Paramedics | 1 | F | | 18-30 | White/British | |
|  |  |  | 4 | F | | 18-30 | White/British | |
|  |  |  | 4 | F | | 18-30 | White/British | |
|  |  |  | 6 | M | | 31-40 | White/British | |
|  |  |  | 16 | M | | 51-60 | White/British | |
|  |  |  | 26 | M | | 61-70 | White/British | |
|  |  | **Sample group** | **Main diagnosis/**  **Relationship** | **Sex** | | **Age** | **Ethnicity** | |
| **CASE 3** |  | Patient | Prostate cancer | M | | 61-70 | White/British | |
|  |  | Carer | Daughter | F | | 51-60 | White/British | |
|  |  | Bereaved carer | Son | M | | 51-60 | White/British | |
|  |  | **Sample group** | **Years since qualification** | **Sex** | | **Age** | | **Ethnicity** |
|  | HEALTHCARE PROFESSIONALS | General practitioners | 19 | F | | 41-50 | | Asian/Asian British |
|  |  |  | 25 | F | | 51-60 | | White/British |
|  |  | Specialist palliative care clinicians | 12 | F | | 31-40 | | White/British |
|  |  |  | 14 | F | | 31-40 | | White/British |
|  |  |  | 18 | F | | 41-50 | | White/British |
|  |  |  | 25 | F | | 51-60 | | White/British |
|  |  | Community nurses | 5 | F | | 18-30 | | White/British |
|  |  |  | 8 | F | | 51-60 | | White/British |
|  |  |  | 10 | M | | 31-40 | | White/British |
|  |  |  | 12 | F | | 31-40 | | White/British |
|  |  |  | 33 | F | | 51-60 | | White/British |
|  |  | Paramedics and ambulance technicians | 2 | F | | 18-30 | | White/British |
|  |  |  | 3 | F | | 18-30 | | White/British |
|  |  |  | 7 | M | | 41-50 | | White/British |
|  |  |  | 10 | F | | 31-40 | | White/British |
|  |  |  | 10 | M | | 41-50 | | White/British |
|  |  |  | 11 | M | | 31-40 | | White/British |
|  |  | Paramedics | 1 | F | 31-40 | White/British | | |
|  |  |  | 2 | F | 31-40 | White/British | | |
|  |  |  | 3 | F | 31-40 | White/British | | |
|  |  |  | 5 | M | 18-30 | White/British | | |
|  |  |  | 6 | F | 41-50 | White/British | | |
|  |  |  | 18 | M | 41-50 | White/British | | |
